# 2797. Antimicrobial Photodynamic Therapy (aPDT) Is Highly Effective Against Multidrug-Resistant HA- and CA- *S. aureus* Strains

**DOI:** 10.1093/ofid/ofad500.2408

**Published:** 2023-11-27

**Authors:** Cristina P Romo_Bernal, Micah Chavez, Sheeny Levengood, Caetano Sabino, Nicolas G Loebel

**Affiliations:** Ondine Research Laboratories, Bothell, Washington; Ondine Research Laboratories, Bothell, Washington; Ondine Research Laboratories, Bothell, Washington; Ondine Research Laboratories, Bothell, Washington; Ondine Biomedical Inc., Bothell, Washington

## Abstract

**Background:**

Methicillin-resistant *Staphylococcus aureus* (MRSA) is often associated with multidrug-resistant (MDR) infections found in healthcare settings, resulting in 48,000 deaths in the US and 1.27 million deaths worldwide each year. *S. aureus* strains are a common component of nasal microbiota and can be disseminated by both patients and healthcare workers, posing a substantial hospital-acquired infection risk. Antimicrobial photodynamic therapy (aPDT) combines the use of a photosensitizer (PS) with a specific wavelength of light to induce photochemical reactions lethal to a broad spectrum of microbes, without resistance induction. The objective of this study was to demonstrate the efficacy of aPDT against clinically-relevant multidrug-resistant *S. aureus* strains using a commercially-available photosensitizer formulation (Steriwave^TM^, Ondine Biomedical Inc., Vancouver, BC).

Resistance profile of MRSA strains

**Figure:**
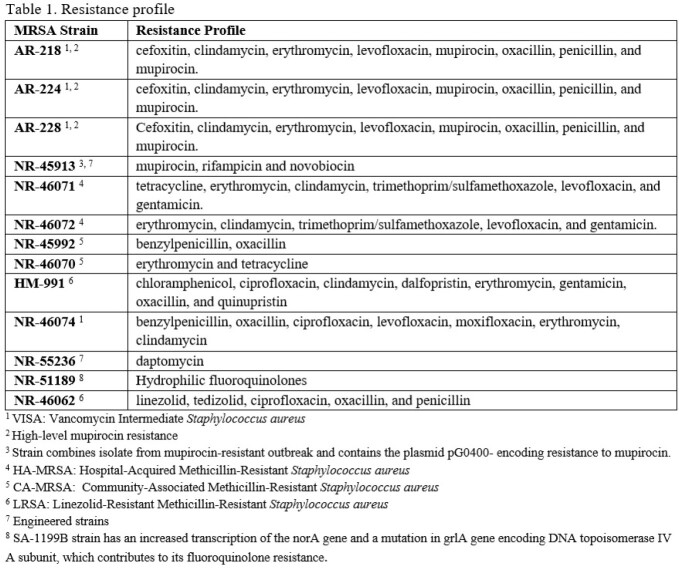

**Methods:**

The MRSA strains used in this study included confirmed resistance to: cefoxitin, clindamycin, erythromycin, levofloxacin, mupirocin, oxacillin, penicillin, rifampicin, novobiocin, tetracyclines, trimethoprim/sulfamethoxazole, gentamicin, chloramphenicol, ciprofloxacin, moxifloxacin, linezolid, tedizolid quinupristin/dalfopristin and mupirocin. aPDT was carried out by exposing planktonic suspensions of each strain to photosensitizer formulations containing either 0.01% MB or Steriwave^TM^ (0.01% MB and 0.25% chlorhexidine gluconate in an aqueous excipient base). Illumination was performed at 670 nm, 150 mW/cm^2^ for 60 s (9 J/cm^2^). Samples were serially diluted and plated on TSA for CFU counting

**Results:**

Relative to untreated controls, aPDT resulted in a minimum reduction of 3log_10_ (99.9%) against all MRSA strains tested, with the commercial Steriwave^TM^ formulation producing an average of 10X greater kills than aqueous methylene blue under the same illumination parameters.

**Conclusion:**

aPDT is highly effective against a variety of clinically-relevant multidrug-resistant *S. aureus* strains in 60 s of treatment. The technique represents a promising alternative to antibiotics in healthcare systems where antimicrobial resistance strategies are important

**Disclosures:**

**Nicolas G. Loebel, PhD**, ONDINE Biomedical Inc.: Board Member|ONDINE Biomedical Inc.: Employee|ONDINE Biomedical Inc.: Stocks/Bonds

